# Effectiveness of Light-Quality and Dark-White Growth Light Shifts in Short-Term Light Acclimation of Photosynthesis in *Arabidopsis*

**DOI:** 10.3389/fpls.2021.615253

**Published:** 2022-01-03

**Authors:** Elisabeth Hommel, Monique Liebers, Sascha Offermann, Thomas Pfannschmidt

**Affiliations:** ^1^Pflanzenphysiologie, Institut für Biologie, Universität Leipzig, Leipzig, Germany; ^2^Molekulare Pflanzenphysiologie, Institut für Pflanzenwissenschaften und Mikrobiologie, Universität Hamburg, Hamburg, Germany; ^3^Pflanzenphysiologie, Institut für Botanik, Naturwissenschaftliche Fakultät, Leibniz-Universität Hannover, Hanover, Germany

**Keywords:** photosynthesis, state transitions, photosystem II super-complexes, light-quality control, dark-light shifts

## Abstract

Photosynthesis needs to run efficiently under permanently changing illumination. To achieve this, highly dynamic acclimation processes optimize photosynthetic performance under a variety of rapidly changing light conditions. Such acclimation responses are acting by a complex interplay of reversible molecular changes in the photosynthetic antenna or photosystem assemblies which dissipate excess energy and balance uneven excitation between the two photosystems. This includes a number of non-photochemical quenching processes including state transitions and photosystem II remodeling. In the laboratory such processes are typically studied by selective illumination set-ups. Two set-ups known to be effective in a highly similar manner are (i) light quality shifts (inducing a preferential excitation of one photosystem over the other) or (ii) dark-light shifts (inducing a general off-on switch of the light harvesting machinery). Both set-ups result in similar effects on the plastoquinone redox state, but their equivalence in induction of photosynthetic acclimation responses remained still open. Here, we present a comparative study in which dark-light and light-quality shifts were applied to samples of the same growth batches of plants. Both illumination set-ups caused comparable effects on the phosphorylation of LHCII complexes and, hence, on the performance of state transitions, but generated different effects on the degree of state transitions and the formation of PSII super-complexes. The two light set-ups, thus, are not fully equivalent in their physiological effectiveness potentially leading to different conclusions in mechanistic models of photosynthetic acclimation. Studies on the regulation of photosynthetic light acclimation, therefore, requires to regard the respective illumination test set-up as a critical parameter that needs to be considered in the discussion of mechanistic and regulatory aspects in this subject.

## Introduction

In oxygenic photosynthesis of plant and algae chloroplasts photosystem II (PSII) and photosystem I (PSI) work electrochemically in series. Efficient electron transport from the donor at PSII, water, to the final acceptor at PSI, NADP^+^, therefore, requires a balanced action of both photosystems. The reaction centers possess slightly different absorption maxima of 680 nm for PSII and 700 nm for PSI. Enrichment of either wavelength in the incident light, thus, can cause imbalances in photosystems excitation which in turn reduces the efficiency in photosynthetic energy conversion (Allen and Pfannschmidt, 2000). Many abiotic and biotic influences can lead to variations in the illumination of plants. A number of highly sophisticated regulation mechanisms evolved that acclimate the process of photosynthetic light harvesting to variations in both light intensity and light-quality that can occur at time scales ranging from seconds to minutes as well as from daily to seasonal variations (Kanervo et al., 2005; Walters, 2005; Eberhard et al., 2008).

In dense populations of terrestrial plants one can observe an exponential decrease in light intensity and a concomitant enrichment of far-red light wavelengths both caused by selective absorption of photosynthetically active radiation (PAR) from the top leaves of the canopy (Terashima and Hikosaka, 1995; Dietzel et al., 2008; Johnson and Wientjes, 2020). Far-red light enriched environments typically lead to relative over-excitation of PSI and a subsequent oxidation of the intermittent electron carriers such as plastoquinone (PQ). Sudden light flashes or long-term exposure to direct sun-light caused by leaf movement through wind or growth can induce the opposite situation in which preferential excitation of PSII creates a more reduced state of the PQ pool. The PQ oxidation at the cytochrome b_6_f complex, an electron transport complex functionally placed between PSII and PSI, is the slowest and, therefore, rate-limiting step of the photosynthetic electron transport. It, thus, represents an ideal sensor for environmental fluctuations (Pfannschmidt, 2003). Indeed, the reduction/oxidation (redox) state of the PQ pool was found to be a key regulator of important photosynthetic acclimation processes including short-term and long-term acclimation responses, such as state transitions and photosystem stoichiometry adjustment (Dietzel et al., 2008; Johnson and Wientjes, 2020 (Goldschmidt-Clermont and Bassi, 2015).

State transitions represent a short-term regulation mechanism for excitation energy redistribution in which the relative antenna cross section of PSII and PSI is modulated through selective phosphorylation of the light harvesting complex of PSII (LHCII) (Allen and Forsberg, 2001). Upon preferential excitation of PSII the PQ pool becomes more reduced. This activates by still unclear mechanistic means the thylakoid-bound kinase STN7 (Bellafiore et al., 2005) that phosphorylates the PSII-bound LHCII. This phosphorylation induces a lateral migration of parts of the LHCII to PSI, thereby reducing the PSII antenna cross section (and its photon absorption capability) and enlarging that of PSI with the goal to redirect more light energy to the rate limiting PSI (state 2) (Rochaix, 2013; Pan et al., 2018). In the opposite case oxidation of the PQ pool was observed to be accompanied by an inactivation of STN7 and a constitutively active thylakoid bound phosphatase PPH1/TAP38 (Pribil et al., 2010; Shapiguzov et al., 2010) de-phosphorylates the LHCII bound to PSI inducing a lateral re-migration to PSII (state 1). PSII core proteins are phosphorylated by another kinase called STN8 that is not under direct control of the PQ pool (Bonardi et al., 2005; Vainonen et al., 2005). It, however, displays a partial substrate overlap with STN7 and is involved in many aspects of photosynthetic regulation that are related to STN7-controlled functions. Understanding photosynthetic regulation by phosphorylation, therefore, requires considering both kinase activities (Rochaix, 2013). Recent studies uncovered that phosphorylated Lhcb1 and Lhcb2 proteins perform different roles during state transitions with Lhcb2 being the dominant protein in this process (Pietrzykowska et al., 2014; Crepin and Caffarri, 2015; Longoni et al., 2015).

Our understanding of the mechanistic steps triggering state transitions is highly complicated by the fact that the linear electron transfer function is not reflected in the structure of the photosynthetic apparatus. The thylakoid membrane system in which the photosystems reside possesses a highly organized three-dimensional structure and can be distinguished into grana membranes with tightly appressed membrane stacks and the interconnecting stroma lamellae. The precise structure of the thylakoid membrane system is still under investigation (Pribil et al., 2014; Kowalewska et al., 2016), however, it is commonly accepted that PSII and its LHCII are located within the grana stacks while PSI (and also the ATPase) are located in grana margins and stroma lamellae since their stromal protein extrusions sterically prevent a presence within the appressed grana membrane stacks (Dekker and Boekema, 2005). Movement of LHCII complexes between PSII and PSI would not only require lateral migration in a membrane but also a movement between grana and stroma lamellae sections. Such directed migration is mechanistically difficult to explain regarding the complex structure of the thylakoid membrane and its crowding with embedded protein complexes that cause steric hindrances (Kirchhoff et al., 2011). The topic of LHCII migration during state transitions is still not fully understood and alternative models for these movements (and thus for state transitions) are proposed in which not only the LHCII but also the photosystems move in order to generate PSII/LHCII/PSI hyper-complexes (Tikkanen et al., 2011).

The structure of the thylakoid membrane system is not fixed but highly dynamic. Phosphorylation of LHCII complexes and/or PSII core proteins within the grana stacks induces a (partial) de-stacking of the membrane structure probably by introducing negative charges on both sides of the membrane stack that repel each other (Allen, 1992). It is important to note that the LHCII kinase STN7 likely cannot enter the grana due to an extruding stromal domain (Rochaix, 2013). Phosphorylation of LHCII, thus, can occur only at the grana margins where the kinase is located. LHCII phosphorylation and subsequent grana membrane de-stacking, therefore, can happen only step-wise from outside to inside and its kinetic depends on the number of available LHCII complexes that reach the STN7 kinase domain. Similar structural constraints may occur also in PSII core phosphorylation events that are suggested to play a role in antenna dissociation and core monomerization (Puthiyaveetil and Kirchhoff, 2013).

Functional PSII complexes within grana stacks typically form dimers that associate with various amounts of LHCII trimers generating so-called PSII super-complexes. The largest association stably isolated after detergent treatment consists of two PSII core (C) complexes with each having one strongly (S) and one moderately (M) bound LHCII trimer generating the C_2_S_2_M_2_ complex (Caffarri et al., 2009). *In vivo* larger associations might be possible. Partial dissociation of this C_2_S_2_M_2_ complex generates smaller super-complexes while aggregations of it create so-called mega-complexes that can even form para-crystalline structures in the grana membranes (Dekker and Boekema, 2005). Upon a state 1-to-state 2 transition a decrease in C_2_S_2_M_2_ complexes and a concomitant increase of C_2_S_2_M_1_ complexes has been observed that suggest an involvement of the M complexes in state transitions (Kouril et al., 2005). In addition, extra LHCII trimers with only very loose contact to the PSII core are located between the super-complexes and in the grana margins providing further candidate trimers that potentially migrate during state transitions (Kouril et al., 2013).

Phosphorylation of PSII core and LHCII trimers is a major determinant of the highly complex arrangement of the photosynthetic apparatus and the thylakoid membrane structure, but the precise sequence of events is far from being clear. We recently could demonstrate that light-quality shifts that either reduce or oxidize the photosynthetic electron transport chain do have pronounced effects not only on STN7 activity and state transitions but also on the formation and the release of PSII super-complexes (Dietzel et al., 2011). These changes in PSII super-complex accumulation occur in the same time range as state transitions and appear to be a limiting factor for them as the amount of PSII super-complexes correlates inversely with the speed of state transitions. According to current data the PSII super-complex release (and presumably the release of PSII super-complexes attached to each other) starts most likely with the phosphorylation of CP43, a core protein of PSII. Stn7/Stn8 double mutants that lack any PSII/LHCII phosphorylation cannot release PSII super-complexes (and likely also structures of higher order such as mega-complexes) and do not display any state transitions. Conversely, in mutants that cannot form PSII super-complexes state transitions run faster. This effect is likely caused by a faster LHCII trimer phosphorylation that is required for state transitions because of higher mobility of the trimers in the membrane (Damkjaer et al., 2009; Dietzel et al., 2011; Garcia-Cerdan et al., 2011). The trimer identity and role of LHCII phosphorylation during PSII remodeling is, however, still under debate as also alternative models have been presented proposing that the so-called extra LHCII trimers are phosphorylated and migrate to PSI during a state 1-to-state 2 transition (Wientjes et al., 2013). Concomitant with that the LHCII trimers in PSII super-complexes were found to be phosphorylated but did not induce a release of the complexes. It was concluded that phosphorylation is not sufficient for a release of PSII super-complexes and that these are stable during a state 1-to-state 2 transition (Wientjes et al., 2013). Recent studies revealed that also the activity of the chloroplast acetyltransferase NSI is required for state transitions and thylakoid membrane remodeling (Koskela et al., 2018, 2020). While the precise mechanistic involvement of protein acetylation requires further investigation these studies reveal that photosynthetic light acclimation is not only dependent on phosphorylation of LHCII proteins but may involve additional post-translational modifications of thylakoid membrane proteins.

Research on processes involved in state transitions typically is done in the laboratory on plants grown under defined light conditions that induce either reduction or oxidation of the PQ pool, respectively. These conditions may be induced by technically slightly different test set-ups, but not much attention has been given yet to such details. We were wondering whether illumination set-ups with small different technical settings but with comparable effects on the PQ pool redox state do induce equivalent physiological effects on photosynthetic acclimation. To this end, we compared light-quality shifts and dark-white light shifts in their effectiveness on state transitions, photosynthetic antenna phosphorylation and PSII super-complex formation.

## Materials and Methods

### Plant Material

*Arabidopsis thaliana* var. Columbia-O (Col-0) plants were grown on soil for 14 days under long day (LD) conditions (16 h light/8 h dark) at 50 μmol m^–2^ s^–1^ white light (Lumilux “Cool White” L 18W/840, Osram) at 23°C and 60% humidity before subsequent light treatments. Plants subjected to long-term light-quality treatment (6 days under continuous PSI- or PSII-light) were grown for 5 days in LD conditions followed by 3 days of continuous WL (to adapt the plants to the continuous illumination) matching a final age of 14 days. Plant material was grown in several parallel pots allowing to split plant sets for individual light treatments after a common initial growth phase. All growth experiments and their subsequent physiological and molecular characterizations were done with at least three independent biological replicates.

### Illumination Set-Ups and Thylakoid Membrane Isolation

After 14 days of pre-cultivation under LD conditions (if not indicated otherwise) plants were subjected to various illumination set-ups for the indicated time periods. White light (WL) treatments were performed under the identical light sources as used for pre-cultivation. The technological set-up for PSI- and PSII-light treatments was identical to that described in Wagner et al. (2008) and Dietzel et al. (2011). For better understanding and interpretation of the physiological effects the emission spectra of the WL, PSI- and PSII-light sources within the growth chambers were recorded with a spectrometer (LI-180, LI-COR Biosciences). These spectra are given in the Supplementary Figure S1. After the respective light treatments (compare Table 1) plant material was harvested directly under the light source and put immediately into ice-cold homogenization buffer followed by isolation of thylakoid membranes as described (Järvi et al., 2011). Chlorophyll concentration of resulting samples was determined according to Porra et al. (1989). Thylakoid samples isolated for BN-PAGE analysis were immediately processed after isolation. Aliquots from these samples were frozen for western-immune-blot analyses after SDS-PAGE and kept at −80°C until further use.

**TABLE 1:** Summary of illumination programs and the corresponding physiological questions investigated by these approaches.

Abbreviation	Illumination program	Physiological state in question
**Control**		
Dark	14 d of growth in 16 h WL/8 h dark; harvest under safe green light before beginning of next light period	State of PS at the end of a dark period in a standard dark-light growth schedule (state 1 as in Wientjes et al., 2013).
**Light-quality shifts**		
Dark - PSII 50	14 d of growth in 16 h WL/8 h dark + 50 min PSII-light	Induction of state 2 by 50 min of PSII-light instead of WL.
Dark - PSII 50 - PSI 30	14 d of growth in 16 h WL/8 h Dark + 50 min PSII-light + 30 min PSI-light	Induction of state 1 by additional 30 min of PSI-light.
**Dark-white light (WL) shifts**		
Dark - WL 50	14 d of growth in 16 h WL/8 h dark + 50 min WL	State of PS after 50 min of WL following the last dark period (state 2 as in Wientjes et al., 2013).
Dark - WL 50 - Dark 15	14 d of growth in 16 h WL/8 h dark + 50 min WL + 15 min dark	Effect of additional 15 min of dark after 50 min illumination (re-opening of PSII centers).
Dark - WL 2 h	14 d of growth in 16 h WL/8 h dark + 2 h WL	Approval of state 2 after extended illumination with WL.
Dark - WL 2 h - Dark 15 min	14 d of growth in 16 h WL/8 h dark + 2 h WL + 15 min dark	Effect of additional 15 min of dark after extended WL acclimation (re-opening of PSII centers).

*All plants analyzed were 14 days old. For plants subjected to long-term light-quality treatments the pre-growth phase was correspondingly shortened. Abbreviations used: d, days; h, hours; min, minutes; PS, photosynthesis apparatus. Abbreviations for light sources are as given in main text.*

### Room Temperature Chlorophyll Fluorescence Measurements

Time course and degree of Chl fluorescence transients of 14-to-21-day-old WT plants and *stn7* mutants grown under DL conditions subjected to indicated shifts in illumination were detected using a pulse amplitude modulation (PAM)-based fluorometer (Junior-PAM, Walz). The measurements aimed to analyze the general dynamics in the Chl fluorescence changes induced by the indicated light shifts with a special emphasis on the process of state transitions. As reference a standard state transition experiment using the internal monochromatic light sources (blue LED, 450 nm, far-red LED, 730 nm) of the Junior-PAM device was performed. To this end plants in their growth pots were placed under the Junior-PAM detector in a way that Chl fluorescence detection was yielding sufficient intensity, but without using a clamp for the leaf that could influence gas exchange. The distance between the fiber optics probe and the leaf surface was adjusted to 5 mm. After 1 h of dark acclimation *F*_0_ and *F*_*m*_ were determined for calculation of *F*_*v*_/*F*_*m*_ (*F*_*v*_ = *F*_*m*_ − *F*_0_) using the Junior-PAM internal measuring light and saturation pulses. Then, actinic light (blue LED, 45 μmol photons m^–2^ s^–1^) was switched on for 30 min for induction of state 2. At steady state fluorescence in state 2 (*F*_*s*2_) a saturation pulse was given for detection of *F*_*m*2_′. Then, far-red light (Junior-PAM, level 12) was added for further 30 min to induce state 1. When reaching a steady state fluorescence in state 1 (*F*_*s*1_) another saturation pulse was given for detection of *F*_*m*1_′. Reversibility of the light-induced acclimation responses was checked in each replicate by switching off the far-red LED allowing for returning to state 2. Determination of Chl fluorescence transients and state transitions induced by growth light sources were done essentially in the same manner as described for the LED-driven reference experiment, but using shifts between the growth lights instead of the internal LEDs. In light quality shift experiments state 2 was induced in 1 h dark-acclimated plants by 50 min illumination with PSII-light, while state 1 was induced by subsequent illumination with PSI-light for 30 min. Reversibility of state transitions in these conditions was checked by a switch back to PSII-light for each replicate. Dark-light shift experiments were performed analogously starting with 1 h dark-acclimation, followed by a 50 min illumination period with white light to induce state 2, followed by a 30 min shift to darkness to induce state 1. Again, reversibility of state transitions was checked by another 30 min of illumination with white light. Calculations of state transition parameters for all light set-ups are given in legend of Table 2.

**FIGURE 1:**
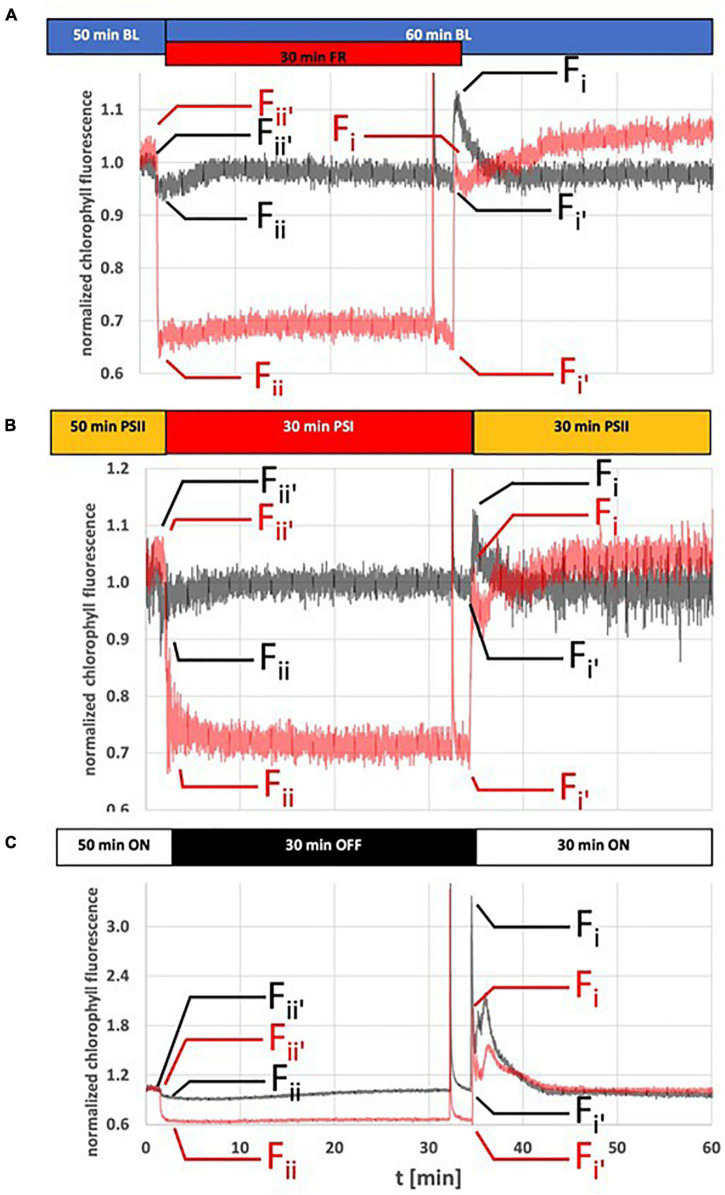
Dynamics of Chl fluorescence changes in response to light quality or dark-light shifts. *Arabidopsis* WT plants (gray traces) and *stn7* mutants (red traces) were grown for 14 days under LD conditions and then used for Chl fluorescence measurements using a pulse amplitude modulated fluorometer. **(A)** Reference measurement using internal LED based light sources of the measurement device for induction of state transitions. **(B)** Chl fluorescence changes caused by shifts between the PSI- and PSII-light sources at equal PAR. **(C)** Chl fluorescence changes caused by dark-white light shifts. Duration of and shifts in illumination are indicated by horizontal bars on top of the fluorescence traces. All experiments started with a 60 min dark-adaptation followed by the light regime indicated in the figure. A saturation light pulse was given 2 min before a change in light condition. Only the pulse in state 1 is visible at this magnification of the figure. *F*_*ii*_′, *F*_*ii*_, *F*_*i*_′, and *F*_*i*_ values were taken at indicated time point (small arrows) in order to calculate *F*_*r*_ according to the protocol by Haldrup et al. (2001). Chl fluorescence is given as normalized values. The figure depicts representative results. Each experiment was repeated at least three times with three different biological samples. For understanding of Chl fluorescence nomenclature compare legend of Table 2. Full data sets for all graphs including a full representation of saturation light pulses are available in Supplementary Figure S2.

### Detection of Thylakoid Membrane Proteins and Their Phosphorylation States

Total accumulation and phosphorylation state of proteins or complexes were determined by western-immune-analyses using protein-specific antisera directed against D1, D2, CP43, Lhcb1 (Agrisera No. AS05084, AS06146, AS111787, AS01004) or an anti-phospho-threonine antibody (Cell Signaling Technology^®^) and the enhanced chemiluminescence (ECL) assay. Indicated amounts of samples were separated by either denaturing SDS-PAGE or BN-PAGE and transferred to nitrocellulose membrane (^®^PROTAN Whatman) by semi-dry-western blotting using standard protocols (Sambrook and Russel, 2001). ECL visualization of primary antisera was done using suitable secondary anti-sera fused to horseradish peroxidase and a Chemi-Doc-™MP Imaging System (BioRad Laboratories). Recording of large differences in signal intensities occurring on one and the same membrane (especially those between phosphorylated core and light harvesting proteins) was done by a series of increasing exposition times in the camera system allowing for respecting the linearity between protein loading and signal intensity. Optimal loading of SDS-PAGE gels with respect to equality of protein amounts between different samples and the linearity between loaded protein amount and resulting ECL signal was systematically determined by serial dilution of WL extracts and determination of the corresponding phospho-threonine signals. Best signal ratios were obtained in the range of 20–30 μg total chlorophyll in the protein extracts and all subsequent experiments were adjusted to this range. In addition, equal loading of each SDS-PAGE gel was tested after the immune-detection using amido-black or Ponceau S staining of the respective membranes.

### BN-PAGE and 2D BN-PAGE

Both BN-PAGE and 2D BN-PAGE were performed essentially as described earlier (Dietzel et al., 2011). Thylakoid sample volumes corresponding to 20 or 30 μg chlorophyll were used for solubilization of thylakoid membranes by adding β-dodecyl-maltoside (β-DM) to a final concentration of 1% (w/v). Samples were separated on 0.75 mm × 12 cm × 18 cm native 5–12% acrylamide gradient gels. The BN gel run was performed for 15 h (over-night) at 70 V in a cold chamber at 4°C. The voltage was then adjusted to 250 V until the run was finished. The cathode blue buffer was replaced by colorless buffer after 2/3 of the gel run. The BN gels were photographically documented, disassembled and cut into slices isolating the different sample lanes. These slices were incubated in denaturing Laemmli buffer [138 mM Tris/HCL pH 6.8, 6M urea, 22.2% (w/v) glycerol, 4.3% (w/v) SDS, 5% (v/v) 2-mercaptoethanol] for 1 h in a Petri dish under gentle shaking, loaded on a denaturing 6–12% acrylamide SDS gradient gel and separated over night at 12 mA. Finally, the gels were silver-stained following standard protocols (Sambrook and Russel, 2001) and photographically documented.

## Results

### Induction and Dynamics of Photosynthetic Acclimation in Response to Light Quality and Dark-White Light Shifts

Recent studies reported 77K fluorescence emission spectra indicating that both, light-quality and dark-white light shifts, induce a significant lateral movement of the mobile LHCII antenna between PSII and PSI resulting in considerable changes of the respective antenna cross sections. The state transition deficient *Arabidopsis* mutant *stn7* was devoid of such changes indicating that the differences in the observed 77K spectra of WT and mutant were caused by short-term antenna movements, i.e., state transitions (Dietzel et al., 2011; Koskela et al., 2018). The 77K measurements, however, provide only static start and end point comparisons missing the dynamics of the respective acclimation responses. In order to follow the course of photosynthetic acclimation induced by light-quality or dark-white light shifts we recorded room temperature (RT) Chl fluorescence transients of plants subjected to corresponding growth light shifts. The *Arabidopsis* state transition mutant *stn7* was used as reference throughout all experiments in order to distinguish between the impact of energy dependent quenching (qE) and state transitions (qT). The impact of photoinhibition-dependent quenching (qI) was regarded as negligible since only weak actinic light was used for excitation in these experiments.

As technical reference for the two illumination set-ups studied we recorded Chl fluorescence changes of WL-grown wild-type control plants (WT) subjected to standard LEDs providing monochromatic blue and far-red light as typically used in PAM-derived Chl fluorescence measurements (for detection of state transitions) (Figure 1A). Illumination with blue light as actinic light source was used to induce a stable steady state fluorescence resulting in state 2 in WT plants (dark gray traces, Figure 1A). Additionally applied far-red light resulted in a sudden fluorescence drop caused by enhanced excitation of PSI that accelerates the withdrawal of electrons from the inter-photosystem transport chain leading to the oxidation of the PQ pool. Subsequently a sigmoidal increase in fluorescence was observed (corresponding to a state 2–state 1 transition) that reached the steady state (state 1) after ∼10 min, a time frame comparable to earlier reports (Dietzel et al., 2011; Koskela et al., 2018). After switching off the far-red light source the Chl fluorescence values exhibited a sharp increase followed by rapid quenching reaching a steady state after ∼10 min corresponding to a state 1–state 2 transition. This sharp fluorescence peak is caused by the sudden over-excitation of PSII due to the decelerated removal of electrons from the PQ pool. Notably, the steady state fluorescence in state 2 and state 1 stabilized at the same values indicating the balancing effect of state transition in electron transport. Performing the same light quality shifts with the *stn7* mutant resulted in a completely different response pattern (red traces, Figure 1A). Far-red illumination of the mutant caused a much stronger drop in fluorescence than in WT which is most likely caused by the large immobile PSII antenna since the mutant is unable to perform a transition to state 2. Unlike the WT the mutant was unable to return to the steady state fluorescence level, although we observed a weak increase within the first 15 min that may indicate that the mutant was still able to perform some minor antenna re-arrangements of unknown origin. Switching off the far-red light source resulted in a jump of the fluorescence signal back to the original values followed by a slow further increase again suggesting some minor re-arrangements in the antenna or the photosynthetic electron transport chain. It must be noted that the fluorescence values of WT and mutant were normalized to the values in state 2. Since Chl fluorescence measurements provide only relative values, it is not possible to compare Chl fluorescence values in absolute terms. Likely the state 2 fluorescence in the *stn7* mutant is higher than in WT but since an internal control is not available this could not be quantified.

Subjecting WT plants to shifts between PSII- and PSI-growth lights resulted in Chl fluorescence changes highly comparable to the LED based system (Figure 1B). The fluorescence drop after shift from PSII- to PSI-light was somewhat more pronounced than with the addition of far-red LED light, therefore the subsequent fluorescence rise took a bit longer in total time, but fully recovered. A shift back to PSII-light induced a slightly stronger fluorescence peak that, however, was fully quenched in a similar time range as in the LED-based system. Also the *stn7* mutant displayed Chl fluorescence changes in response to the light shifts which were highly similar to those in the LED-based system. In sum, the obtained data indicate that shifts between the PSI- and PSII-growths are able to effectively induce state transitions.

Next, we performed corresponding experiments using dark-white light shifts for manipulation of photosynthetic electron transport (Figure 1C). Dark-acclimated wild-type plants were illuminated for 30 min with white light in a comparable intensity as with the PSII-light source until the plants reached a stable steady state fluorescence. Shifting plants to dark conditions induced a drop in Chl fluorescence that was followed by a slow sigmoidal increase in fluorescence that reached the steady state (state 1) after ∼30 min. A shift back to white light induced a sharp fluorescence rise that was quickly quenched by the light activation of photochemical and non-photochemical quenching (NPQ) processes. The same experiments done with the *stn7* mutant resulted in a significant drop in fluorescence after shift to darkness. The mutant was unable to return to the starting level in Chl fluorescence, thus the slow sigmoidal return observed in WT can be regarded as a transition to state 1. A shift back to white light induced a fluorescence rise that was considerably smaller than in WT followed by rapid quenching comparable to the WT control.

The dark-to-white light shift is used in standard experimental photosynthesis set-ups to determine the effectiveness of photochemical (PQ) and non-photochemical quenching (NPQ) processes. Since these processes occur in parallel their relative contribution to the overall Chl fluorescence quenching is difficult to quantify, but for determination of state transition dependent quenching (qT) an established protocol exists. This quantification of qT requires saturation light pulses that close the PSII reaction centers and indicate their relative antenna size in either state 1 or state 2 (Haldrup et al., 2001). However, this type of protocol functions properly only under conditions in which the excitation energy of the actinic light source remains stable during measurement without inducing differences in energy-dependent quenching (qE). Since dark-to-WL shifts include intrinsic differences in actinic light intensity a different way of quantification for the contribution of state transitions under these conditions was required. To this end, we determined state transitions in two different ways according to published protocols (Haldrup et al., 2001) as (i) relative change in maximal fluorescence in state 1 and state 2 (qT) and as (ii) relative Chl fluorescence change (*F*_*r*_) that describes the relative difference of fluorescence in state 2 and state 1 upon changes between either PSI- or PSII-light (Haldrup et al., 2001). In order to eliminate the contribution of energy dependent quenching in these calculations we determined all values in parallel for WT and the state transition mutant *stn7* (both grown and measured under identical conditions). Then we subtracted the values of the mutant from WT assuming that the remaining values provide an approximation for the contribution of state transitions to the fluorescence changes in the growth light systems (given as qT _*approx.*_ and Δ*F*_*r*_ in Table 2). According to these results we found that in both growth light set-ups, illumination shifts induce state transitions comparable to the LED-based detection system. All plant batches were in a comparable viability state as indicated by highly similar *F*_*v*_/*F*_*m*_ values (see Table 2) excluding the possibility of interference by stress responses. The Chl fluorescence traces provided also a rough estimate for the speed of state transitions in the different illumination systems. According to these data the state transitions in the LED-based test system and the PSI- PSII-growth light sources were comparable in speed, while that in the white light-to-dark shift was considerably slower (especially for the state 2-to-state 1 transition). A precise determination of the speed of the state 1-to-state 2 transition remained difficult because of the interfering photochemical quenching and NPQ processes, however, in all cases it is likely faster than the state 2-to-state 1 transition since the steady state levels were reached faster.

**TABLE 2:** Chlorophyll fluorescence parameters in the different light set-ups.

Set-up	*F*_*v*_/*F*_*m*_	qT_1_	qT_1_ approx	qT_2_	qT_2_ approx	*F*_*r*_	Δ*F*_*r*_
Internal LED lights	**WT:** 0.76 ± 0.01 ***stn7:*** 0.79 ± 0.01	**WT:** 0.028 ± 0.008 ***stn7*:** **−**0.025 ± 0.002	**0.053** ± 0.007	**WT:** 0.049 ± 0.022 ***stn7:*** −0.032 ± 0.003	**0.082** ± 0.019	**WT:** 0.67 ± 0.05 ***stn7:*** −0.2 ± 0.02	**0.87** ± 0.087
PSII-/PSI-light system	**WT:** 0.81 ± 0.00 ***stn7:*** 0.77 ± 0.03	**WT:** 0.04 ± 0.02 ***stn7***: 0.01 ± 0.01	**0.05** ± 0.071	**WT:** 0.07 ± 0.013 ***stn7:*** 0.027 ± 0.021	**0.047** ± 0.034	**WT:** 0.47 ± 0.2 ***stn7:*** 0.04 ± 0.08	**0.43** ± 0.11
Dark/white- light system	**WT:** 0.82 ± 0.00 ***stn7:*** 0.77 ± 0.01	**WT:** 0.101 ± 0.014 ***stn7:*** 0.044 ± 0.024	**0.057** ± 0.025	**WT:** 0.166 ± 0.036 ***stn7:*** 0.064 ± 0.017	**0.102** ± 0.036	**WT:** 0.9 ± 0.02 ***stn7:*** 0.6 ± 0.03	**0.3** ± 0.066

*Calculation of Chl fluorescence parameters was done with data obtained in experiments shown in Figure 1. Results are means of three independent biological replicates. Standard deviations are given. Calculation of F_v_/F_m_: F_m_ − F_0_/F_m_. Calculation of qT (state transition dependent quenching) with the saturation pulse method was done separately for the transitions from state 2 to state 1 (qT_1_) and from state 1 to state 2 (qT_2_) as: qT_1_ = (F_m1_ − F_m2_)/F_m1_ and qT_2_ = (F_m1_ − F_m2_′)/F_m1_; with F_m1_, F_m2_ and F_m2_′ as maximum fluorescence yields obtained in stable state 1 and 2, respectively (compare Supplementary Figure S1). Approximation of state transitions in the light quality and dark-light shift systems is calculated as: qT_n approx_ = qT_n__WT_–qT_n__stn7_. Calculation of F_r_ according to Haldrup et al. (2001): F_r_ = [(F_i_′ − F_i_) − (F_ii_′ − F_ii_)]/(F_i_′ − F_i_) with F_i_ and F_ii_ as fluorescence yield in state 1 or 2, and with F_i_′ and F_ii_′ as fluorescence yield in state 1 or 2 directly after illumination shift. Approximation of state transitions on base of F_r_ in WT and stn7 is given as: ΔF_r_ = F_r__WT_ − F_r__stn7_.*

### Phosphorylation Changes Induced by Light-Quality or Dark-Light Shifts

**FIGURE 2:**
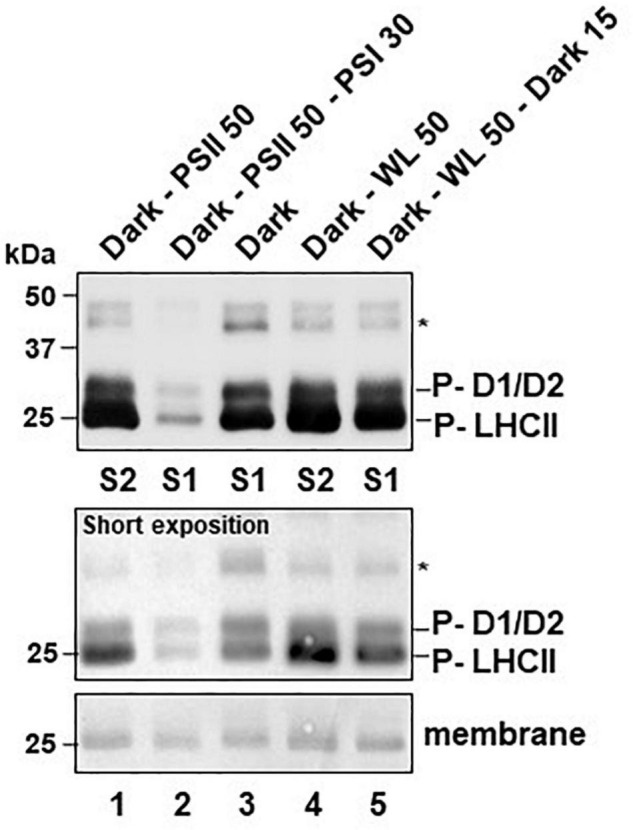
Effects of light-quality or dark-white light shifts on phosphorylation state of thylakoid membrane proteins. Isolated thylakoid protein samples corresponding to 20 μg total chlorophyll were separated by SDS-PAGE. For detection of phosphorylation state proteins were transferred to a nitrocellulose membrane *via* western blot and immuno-decorated using an anti-phospho-threonine antiserum (Cell Signaling Technologies). Labeling was done according to published work (Fristedt et al., 2009; Samol et al., 2012). This phospho-immuno-detection was done once to confirm the state transition results from the Chl fluorescence data and produced results that are in full accordance to earlier studies. A comprehensive statistical treatment of data including triplicate independent biological repetitions, therefore, was regarded as negligible. Relative changes in signal intensities can be sufficiently estimated by comparison to the loading controls given by the respective amido-black stained membranes at the migration front of LHCII trimers (membrane). Additional loading controls are given in Supplementary Figure S3. Sizes of marker proteins are given in the left margins in kDa. Signals from phosphorylated CP43 (labeled by asterisk) and another unidentified protein were very weak requiring long exposition times to be visualized. Signals from phosphorylated D1, D2 and LHCII (P-D1/D2, P-LHCII) were reaching saturation under such conditions and a second short exposition time of the same membrane is given (short exposition) as control. All plants were grown for 2 weeks under LD conditions and treated as indicated before sampling. Dark-PSII 50: 50 min PSII-light after dark. Dark-PSII 50–PSI 30: 50 min PSII-light after dark followed by 30 min of PSI-light. Dark: Control at the end of the last dark period. Dark-WL 50: 50 min WL after dark. Dark-WL 50 - Dark 15: 50 min WL after dark followed by 15 min dark. The respective state induced by the illumination program is indicated at the bottom (S1: state 1; S2: state 2).

In order to obtain independent experimental proof for the effectiveness of the two growth light systems in inducing state transitions we determined the phosphorylation state of thylakoid membrane proteins in the different illumination set-ups by standard procedures (Figure 2). To this end *Arabidopsis thaliana* plants were grown for 2 weeks and subjected to various illumination protocols using light-quality (PSI- or PSII-light) or dark-white light (WL) shifts (explained in Table 1). 50 min illumination with PSII-light after the last dark phase induced a strong phosphorylation of LHCII while additional 30 min of PSI-light resulted in a very low phosphorylation state (Figure 2, P-LHCII, lanes 1–2). The phosphorylation state of control plants harvested at the end of the last dark period was found to be low, but apparently stronger than after the PSI-light treatment (Figure 2, lane 3). When we replaced the PSII-light by WL we observed a strong LHCII phosphorylation that appeared to be slightly stronger than that after PSII-light illumination. Returning such samples for 15 min to the dark partly reversed the phosphorylation state of LHCII (Figure 2, P-LHCII, lanes 4, 5). For PSII core proteins D1, D2 and CP43 we observed similar patterns in phosphorylation changes with the exception that the phosphorylation state was found strongest in the dark control (Figure 2, top panel, lane 3). 50 min of PSII-light slightly weakened this phosphorylation state, but additional 30 min of PSI-light led to almost complete de-phosphorylation. Illumination of plants with 50 min of WL resulted in a comparable effect as with PSII-light and additional 15 min in the dark resulted in a very minor further decrease (Figure 2, top panel, lanes 4, 5).

The observations on LHCII phosphorylation are consistent with earlier reports (Bellafiore et al., 2005; Bonardi et al., 2005; Pesaresi et al., 2009; Tikkanen et al., 2010) and indicate that light-quality shifts as well as dark-light shifts are able to induce LHCII phosphorylation changes that are required for state transitions, i.e., strong LHCII phosphorylation after illumination with PSII-light or WL; and LHCII de-phosphorylation after illumination with PSI-light or transfer to the dark. Control experiments indicated that neither the total protein composition of the thylakoid membrane fractions (Supplementary Figure S3A) nor the accumulation of selected PSII proteins (Supplementary Figure S3B) displayed major changes in response to the applied light shifts. Most importantly, Lhcb1 (one of the major LHCII proteins) remained fully stable under all tested conditions (Supplementary Figure S3B, bottom panel) indicating that the observed variations in phosphorylation state of the antenna (Figure 2) are not caused by differences in protein accumulation. This indicates that both light set-ups are suitable to control the activity of the redox-responsive STN7 kinase (and hence are suitable for the control of the PQ redox state). However, the two light set-ups may generate subtle differences in PSII core protein phosphorylation that could be an interesting target for more specific investigations in future.

### Phosphorylation State of Native Photosystem Complexes and Photosystem II Remodeling in Response to Light-Quality and Dark-Light Shifts

**FIGURE 3:**
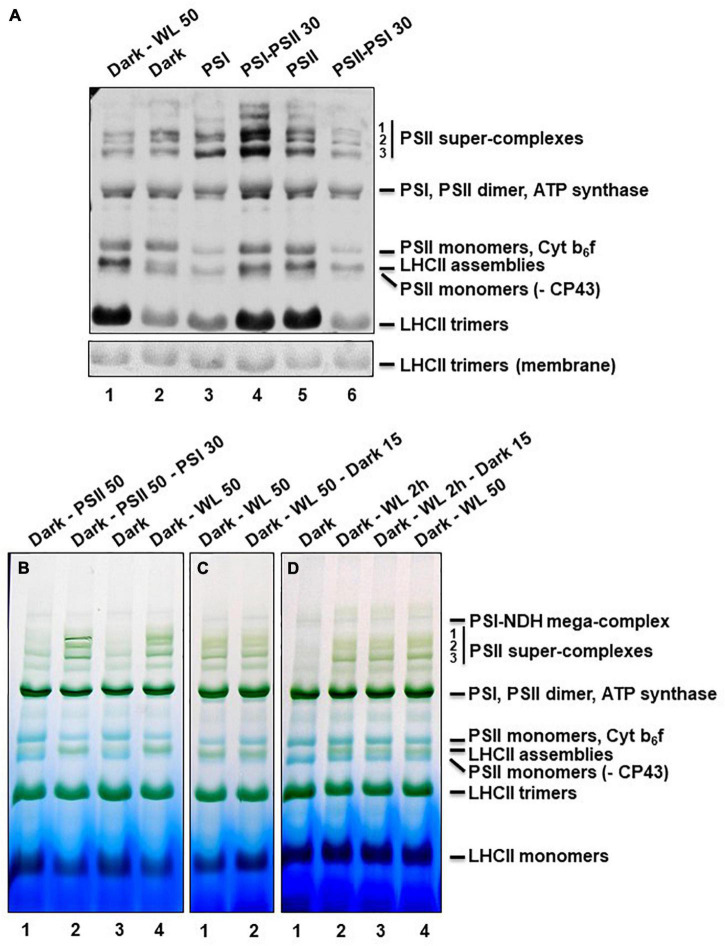
Phosphorylation and assembly states of thylakoid membrane protein complexes after BN-PAGE. **(A)** Thylakoid protein samples corresponding to 20 μg total chlorophyll were separated by BN-PAGE. Material for the first two wells was isolated from plants grown under LD conditions for 2 weeks. Dark-WL 50: 50 min illumination with WL after dark period. Dark: Sample harvested at the end of the night period. All other plants were grown for 5 days under LD conditions, afterward shifted for 3 days to continuous white light and subsequently grown for 6 days under the light-quality regimes indicated on top (matching in total 2 weeks of growth). These samples served as control allowing for comparison with results published earlier (Dietzel et al., 2011). PSI: 6 days PSI-light; PSI-PSII 30: 6 days PSI-light followed by 30 min PSII-light; PSII: 6 days PSII-light; PSII-PSI 30: 6 days PSII-light followed by 30 min PSI-light. The protein complexes separated by the BN-PAGE were denatured in gel by incubation in Laemmli buffer and transferred to a nitrocellulose membrane *via* western blot. Phosphorylation state of thylakoid membrane proteins was detected by incubation with anti-phospho-threonine antibodies. The amido-black stained membrane at the height of LHCII trimers is shown as loading control. Bands were labeled as described (Järvi et al., 2011). The experiment was repeated three times with results showing only marginal variations, thus one representative result is given. Note that the phosphorylation states of the free LHCII trimers correspond well to those shown in Figure 2. **(B–D)** Thylakoid protein samples corresponding to 30 μg total chlorophyll were separated by BN-PAGE. Material separated on the same gel always was isolated from the same growth batch of plants that were all grown under LD conditions for 2 weeks prior to the different short-term illumination treatments indicated on top of each well. Dark samples were harvested at the end of the night period and served as control. All material was harvested directly under the respective light source. **(B)** Dark - PSII 50: 50 min PSII-light after dark; Dark - PSII 50 - PSI 30: 50 min PSII- light after dark followed by 30 min PSI-light; Dark: control at end of dark period; Dark - WL 50: 50 min WL after dark (same in panels **(B)** and **(C)**). **(C)** Dark - WL 50 - dark 15: 50 min WL after dark followed by a 15 min shift into dark. **(D)** Dark - WL 2 h: 2 h WL after dark; Dark - WL 2 h - dark 15: 2 h WL after dark followed by a 15 min shift into dark. Panels **(B–D)** are from individual gels each and display representative results. Each experiment was done at least three times. Bands were labeled (right margin) as described (Dietzel et al., 2011; Järvi et al., 2011). As common control Dark - WL 50 was included in all gels.

Assessments of the phosphorylation state of PS proteins after denaturing SDS-PAGE determines the phosphorylation state of the total fraction of a given protein, but cannot distinguish between different association states of native PS complexes in the membrane. Since state transitions and PSII core protein phosphorylation are known to be also connected to PSII remodeling events we determined the phosphorylation state of the diverse PS complexes after mild solubilization and blue native (BN)-PAGE as reported earlier (Dietzel et al., 2011). Strongest changes in phosphorylation states could be observed for free LHCII trimers and (to a minor degree) for so-called LHCII assemblies (Figure 3A). As in the denaturing approach, illumination with WL and PSII-light (the latter as long-term or short-term application) resulted in a high phosphorylation state while dark or PSI-light illumination induced low phosphorylation states. Further significant differences in phosphorylation states were found in PSII monomers and super-complexes (Figure 3A). The phosphorylation signal of PSII-super-complexes in response to PSII-light was found to be much more pronounced than after PSI-light exposure confirming earlier studies (Dietzel et al., 2011). WL treatment, however, was less effective and could not induce the same strong phosphorylation response as PSII-light (especially after the short-term light shift, Figure 3A, lane 4). The signal intensity in the WL samples was more comparable to that from the dark and PSI-light treated samples. The results indicate that PSII-light and WL illumination induce also at the native level a significant phosphorylation of LHCII complexes confirming the effectiveness of both light set-ups in induction of state transitions. Interpretation of the phosphorylation intensities of PSII super-complexes, however, is more difficult since core protein and LHCII phosphorylation contribute to the resulting signal. Furthermore, the signal intensity might be affected by the actual amount of the respective PSII super-complexes.

In order to study such potentially different effects of light-quality and dark-light shifts on PSII super-complexes, thylakoid membrane complexes from *Arabidopsis thaliana* plants grown under corresponding light conditions were analyzed for their assembly states. To this end plant material was harvested under the respective condition, thylakoids were isolated, membranes were solubilized and blue native (BN) PAGE (Figure 3B) was performed as described earlier (Dietzel et al., 2011). For testing the impact of light-quality on PSII super-complex formation, plants at the end of the night period were illuminated 50 min with PSII-light, one sample was harvested and the remaining plants were further illuminated with PSI-light for 30 min (Figure 3B). While in PSII-light plants only small amounts of PSII super-complexes were detectable, PSI-light exposure induced a strong formation of super-complexes within just 30 min. This is in full accordance with earlier data (Dietzel et al., 2011). Control plants harvested in the dark exhibited only minor amounts of PSII super-complexes that appear to be even less abundant than after PSII-light treatment. In contrast, 50 min of WL exposure caused significant PSII super-complex formation, however, less than after PSI-light treatment (especially with respect to the two largest complexes that correspond to C_2_S_2_M_1_ and C_2_S_2_M_2_ complexes) (Figure 3B). Since in the dark much less PSII super-complexes were found than under WL we concluded that these must exhibit a stronger phosphorylation state than the ones detected in the WL sample (compare to Figure 3A, lanes 1, 2). Quantification of the relative changes in phosphorylation signals and Coomassie staining of PSII super-complexes using ImageJ software confirmed that PSII super-complexes from dark samples exhibited stronger phosphorylation than those isolated from samples exposed to 50 min of WL (see Supplementary Table S1). This indicates that the phosphorylation states of PSII super-complexes isolated from the dark and 50 min WL samples are opposing the respective states of the LHCII trimers within the same samples. These data are in line with the observation that dark samples display higher core protein phosphorylation states (compare to Figure 2). Dark-light shifts, thus, induced state transitions comparable to light-quality shifts, but they caused the opposite effect on PSII super-complex formation. In addition, the PSII super-complex formation induced by WL was found to be not reversible when plants were shifted for 15 min back to dark conditions (Figure 3C). Such a dark treatment is commonly used in room temperature Chl fluorescence experiments to re-open all PSII reaction centers and to transfer all antenna complexes back to PSII. Our observations described here strongly suggest that even though all PSII reaction centers might have re-opened a full PSII remodeling is not achieved within 15 min. A longer dark adaptation phase in such kind of experiments, thus, appears to be recommendable. Prolongation of WL illumination to 2 h did not further increase the accumulation of PSII super-complexes indicating that the 50 min WL exposition was already saturating the response (Figure 3D). In sum, we conclude that dark-light shifts induce (at least in part) different mechanisms in PSII super-complex formation than light-quality shifts. Alternatively, the release mechanisms might be not the same. As a side observation we found that regardless of which light treatment was used, a high abundance of PSII super-complexes was always accompanied by a strong accumulation of an additional green band in the BN-PAGE (Figures 3B–D) that most likely corresponds to LHCII assemblies (Chen et al., 2018) suggesting that the same mechanism that causes the PSII super-complex formation is also involved in the agglomeration of LHCII trimers (or *vice versa*).

### Subunit Composition of Photosystem Complexes After Different Light Treatments

**FIGURE 4:**
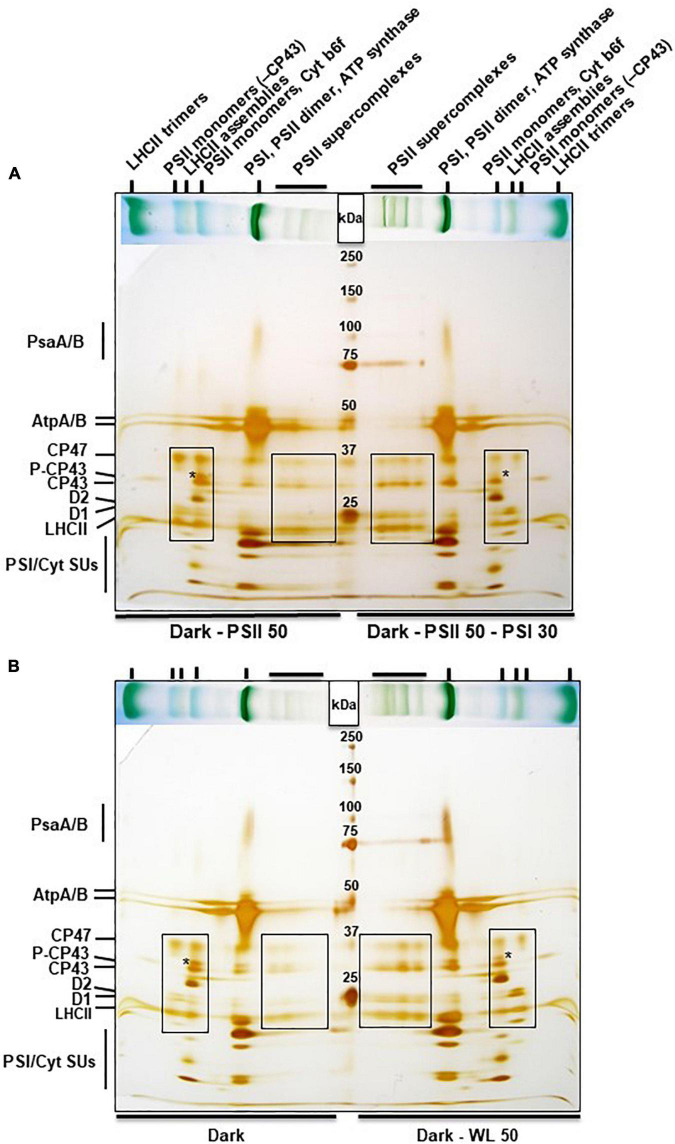
2D BN-PAGE of thylakoid membrane proteins from *A. thaliana* subjected to light quality or dark light shifts. Stripes from BN-PAGE (placed horizontally on top of gels, compare Figure 3) were cut out, denatured in Laemmli buffer and run on a SDS-PAGE as second dimension with a head-to-head orientation. Marker proteins (sizes are given in kDa) were loaded in between. The gels were stained with silver. **(A)** Dark - PSII 50: 50 min PSII-light after dark period; Dark - PSII 50 - PSI 30: 50 min PSII-light after dark period followed by a shift for 30 min to PSI-light. **(B)** Dark: Material harvested at the end of the night phase; Dark - WL 50: 50 min WL illumination after dark phase. Labeling of bands in the BN-PAGE given on top of panel **(A)** is also valid for panel **(B)**, corresponding bands are marked by vertical small lines. Individual protein bands in the second dimension are given in the left margins. Asterisks mark the position of phosphorylated CP43 (right or left from the asterisk, respectively). Broad boxed areas indicate subunits of PSII super-complexes, small boxed areas indicate subunits of PSII monomers. Experiments have been performed three times with results showing only minor variations. Continuous protein bands at 75 kDa in the right half of the gels are leakages from the protein marker.

Next we analyzed the subunit composition of the different protein complexes identified in the BN-PAGE. Lanes with corresponding material separated by BN-PAGE were cut and subjected to a second denaturing dimension as done earlier (Dietzel et al., 2011). Protein subunits contained in the various native complexes were subsequently visualized by silver-staining (Figures 4A,B), and identified according to their reported migration behavior in these gel systems as well as by own confirmation by mass spectrometry (Aro et al., 2005). PSII-light acclimation for 50 min caused a very low accumulation of PSII super-complexes (especially of the two largest ones corresponding to C_2_S_2_M_1_ and C_2_S_2_M_2_ complexes) when compared to the subsequent PSI-light acclimation (see broadly boxed area in Figure 4A). Instead high accumulation of PSII monomers with or without CP43 became visible (small boxed area in Figure 4A). In addition the PSII monomers containing CP43 displayed a very high proportion of phosphorylated CP43 (indicated by an asterisk). PSI-light acclimation caused the opposite reaction with low amounts of monomeric PSII complexes and apparently undetectable traces of phosphorylated CP43. Interestingly, PSII monomers without CP43 were less abundant in these samples suggesting that they may be involved in the formation of the LHCII assemblies.

The same 2D BN-PAGE analysis was performed with material from *Arabidopsis thaliana* plants harvested shortly before the end of the night phase and after 50 min of WL treatment. Material isolated from the dark phase was almost devoid of PSII super-complexes, but displayed a very high proportion of monomeric PSII complexes and a strong accumulation of phosphorylated CP43 (Figure 4B, left side of gel), the latter being consistent with the results presented in Figure 2. 50 min WL treatment induced formation of some PSII super-complexes (mainly the lower two bands). Accumulation of monomeric PSII and of phosphorylated CP43 was less when compared to the dark sample but more when compared to PSI-light acclimated samples (Figure 4A). This indicates that the structure of PSII super-complexes under dark conditions resembles very much that under PSII-light conditions while WL treatment for 50 min induces a condition being somewhat intermediate between PSI- and PSII-light. We conclude that although PSII-light and WL treatments both induce a significant phosphorylation of LHCII they induce different responses in PSII super-complex formation.

## Discussion

Variations in illumination caused by abiotic and biotic factors are one prime source of deleterious effects on photosynthetic efficiency that are counteracted by a multitude of compensation responses such as non-photochemical quenching, state transitions and PSII remodeling events (Morales and Kaiser, 2020). These responses are very complex and typically include structural rearrangements in the photosynthetic apparatus, most of them in the antenna complexes of PSII and PSI (Johnson and Wientjes, 2020). Many details in these antenna rearrangements are not understood yet and, therefore, are investigated in a variety of set-ups. Growth conditions in climate-controlled growth chambers cannot reflect the multitude of variations in the natural environment of a plant, but allow for the analysis of isolated parameters and their effects under standardized conditions. These set-ups, however, are often highly specific and may generate subtle physiological differences that are not apparent on first sight. A careful and detailed analysis, therefore, is highly recommendable for each set-up. Measurement of room temperature Chl fluorescence parameters typically done in a pulse-amplitude modulation (PAM) mode is very informative (Maxwell and Johnson, 2000; Kalaji et al., 2017). However, for these measurements usually the plants are taken out of their growth condition and placed into a measurement room or chamber where they are subjected to highly specific analytic lights. State transition measurements in vascular plants (note that protocols for algae might be different) are often performed with LEDs within the blue range (∼450–470 nm) as actinic light source (compare Figure 1A). This actinic light excites PSII and PSI, drives photosynthetic electron flow and reduces the PQ pool resulting in state 2. Induction of state 1 then is induced by illumination with far-red LEDs (∼730–735 nm) either in replacement or in addition resulting in the oxidation of the PQ pool (compare Figure 1A). Such measurements report on the ability of the plant to perform state transitions, but they do not report whether or not the growth light system is inducing the respective responses. To obtain this information it is required to perform state transition measurements with the growth light sources as analytic lights. This requires to do the measurements directly within the growth unit (compare Figures 1B,C).

Most laboratory growth light systems for plants of at least the last five decades were based on illumination with fluorescent tube lamps, sometimes combined with incandescent bulbs. These light systems are efficient and long-lasting concerning plant growth, but with respect to their light spectrum somewhat limited when compared to natural conditions. When studying photosynthetic light acclimation responses it is, therefore, recommendable to take these specific conditions into consideration. Here we have compared the direct effects of dark-light and PSI-PSII-light shifts of two fluorescent tube-based growth light systems on thylakoid membrane protein phosphorylation, the performance of state transitions and PSII super-complex remodeling in *Arabidopsis* WT plants in order to test how far these two illumination set-ups are comparable.

In principle, one could expect that both conditions, darkness and PSI-light, cause an oxidation of the photosynthetic electron transport chain since in the first case no charge separation can occur while in the second case PSI is working more efficiently than PSII. In contrast, WL and PSII-light should induce a reduction of it including corresponding changes in the PQ pool redox state as both promote charge separation in PSII and subsequent electron transport toward the PQ pool. Consequently, we would expect inactivation or activation of the thylakoid membrane kinase STN7, respectively, and correspondingly we would expect less and more phosphorylation of LHCII. This, indeed, we could observe in the RT Chl fluorescence traces (Figure 1) and the thylakoid membrane phosphorylation state (Figures 2, 3A). It must, however, be noted that the changes in the RT Chl fluorescence traces represent the combined action of several quenching processes and the proportion that state transitions contribute could be determined only by the inclusion of the *stn7* mutant as negative control. This approach provided a reasonable approximation of the extent to which state transitions contribute to the changes in the Chl fluorescence signal observed upon shifts between growth lights. Based on the assumption that the *stn7* mutant performs photochemical quenching comparable to WT we estimate that state transitions contribute between 30 and 50% to the total Chl fluorescence change after the growth light shifts [PSI- to- PSII-light (Figure 1B) and dark- to white light (Figure 1C)]. The proportion of mobile LHCII in vascular plants is estimated to be around just 15–20% (in contrast to 80% in the unicellular alga *Chlamydomonas*) (Rochaix, 2007). Therefore, state transitions are regarded to play only a minor role as quenching process in vascular plants. However, state transitions represent an acclimation response acting preferentially in the low light range (below 100 μE light intensity in vascular plants) since in higher light intensities the LHCII kinase is inactivated (Rintamaki et al., 2000). Our data suggest that in this low light range state transitions may play a more prominent role than anticipated so far in plants, most likely because state transition measurements are typically not done with the growth light source as analytic lights.

**FIGURE 5:**
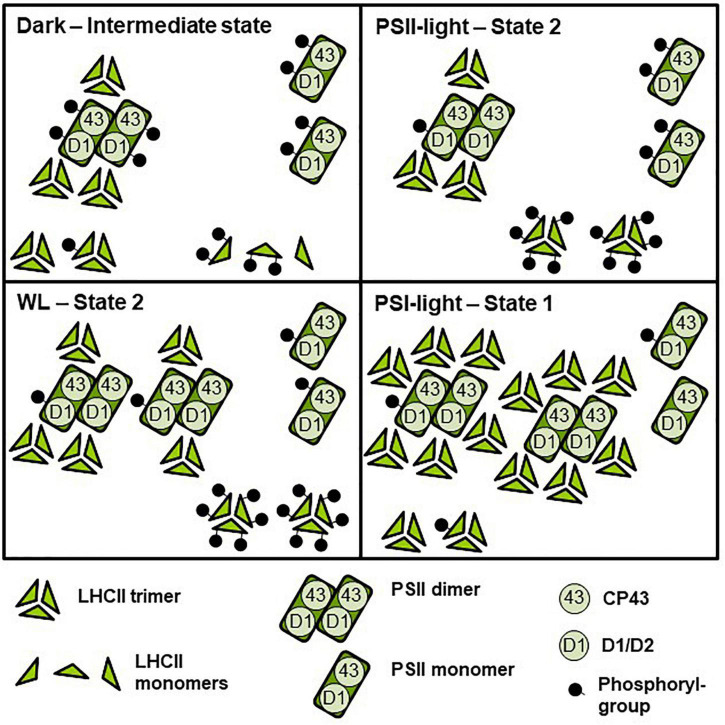
Model depicting effects of light-quality or dark-white light shifts on assembly and phosphorylation states of thylakoid membrane proteins. The simplified scheme integrates the central biochemical data from Figures 2–4 and Supplementary Table S1. Symbol representations are defined at the bottom of the scheme. The number of protein complexes and of phosphoryl-groups per protein complex indicates relative accumulation and degree of phosphorylation between the four different conditions. Dark: Low accumulation of PSII super-complexes, moderate phosphorylation state of PSII-bound LHCII and free LCHII trimers, PSII monomers with strongly phosphorylated CP43. PSII-light – State 2: Low accumulation of PSII super-complexes, PSII monomers with strongly phosphorylated CP43, high phosphorylation state of LHCII trimers. WL: High accumulation of PSII super-complexes (mostly C_2_S_1_ and C_2_S_2_), PSII monomers with low to moderately phosphorylated CP43, very high phosphorylation of LHCII trimers. PSI-light – State 1: Very high accumulation of PSII super-complexes (including C_2_S_2_M_2_ to C_2_S_1_), low phosphorylation state of PSII-bound and free LHCII trimers and of PSII dimers. PSII monomers with largely dephosphorylated CP43. For functional implications of the different assembly and phosphorylation states, see “Discussion” section.

In sum, we regard the two light set-ups as equally effective to induce short term photosynthetic acclimation responses in the antenna, i.e., changes in LHCII phosphorylation and corresponding state transitions. With respect to PSII super-complex formation, however, the two illumination set-ups induce different reactions (Figure 5). The question arises whether this difference in PSII super-complex formation is relevant for state transitions. Analysis of PSII super-complex remodeling induced by light-quality shifts indicated that CP43 phosphorylation in PSII super-complexes (and probably also in mega-complexes) most likely initiates the release of these super-complexes (Dietzel et al., 2011). Indeed, we observed high accumulation of phosphorylated CP43 both in the dark and after PSII-light treatment (Figures 4A,B, asterisks) where only weak super-complex formation is found. In contrast, strong PSII super-complex formation after PSI-light treatment correlated with low accumulation of phosphorylated CP43. More moderate PSII super-complex formation as observed after 50 min WL treatment also correlated with a moderate accumulation of phosphorylated CP43 (Figures 4A,B, asterisks). The phosphorylation states of the native complexes indicate that WL mainly induces the phosphorylation of free LHCII trimers (Figure 3A) that are readily available since in the dark only a minor fraction of PSII super-complexes exists and most LHCII trimers appear to be present as free unbound trimers (Figure 3B). This is in agreement with a recent study reporting that it is a loosely bound (L) LHCII trimer rather than an S or M LHCII trimer from the PSII super-complexes that is involved in the formation of the state-transition induced PSI-LHCII complex (Galka et al., 2012). The STN8 kinase was identified as an enzyme targeting PSII core proteins (Bonardi et al., 2005; Vainonen et al., 2005), however, CP43 phosphorylation dynamics differ from that of other core proteins suggesting the action of another yet unidentified kinase activity (Fristedt et al., 2010). In addition, the degree of PSII phosphorylation was found to differ between genetic accessions of *Arabidopsis* (Yin et al., 2012). Phosphorylation and dephosphorylation of thylakoid photosynthesis proteins depends on a complex interplay of the STN7 and STN8 kinases and their counteracting TAP38/PPH1 and PBCP phosphatases (Wunder et al., 2013). Although already studied in great detail at biochemical and physiological level our understanding of the regulation of these enzymes has still gaps and the involvement of additional enzyme activities such as a kinase activity being responsible for CP43 phosphorylation in the dark might be possible under specific conditions not tested yet. This argues for more systematic studies analyzing phosphorylation/dephosphorylation of thylakoid membrane proteins under a large variety of illumination conditions and in different genetic backgrounds in order to achieve a comprehensive understanding of these processes.

In sum, we conclude that WL illumination does induce a state transition by phosphorylating free LHCII trimers, but at the same time it induces a moderate formation of PSII super-complexes (and a concomitant de-phosphorylation of them) which is in contrast to PSII-light illumination (Figures 2–4), which induces strong phosphorylation of PSII super-complexes (and its subsequent partial release) generating more free LHCII trimers (Figure 5). Some highly phosphorylated PSII super-complexes appear to remain stable suggesting that not alone the phosphorylation state but also other determinants such as crowding of the membrane may have an impact on the release (Figure 3; Dietzel et al., 2011; Kirchhoff, 2014). PSII-light, thus, can induce migration of bound and free LHCII trimers while WL likely induces only that of free LHCII trimers which may explain differing observations (Dietzel et al., 2011; Wientjes et al., 2013). Interestingly, since both light sources are able to induce state transitions this suggests that LHCII complexes that migrate during state transitions may potentially originate from different pools of LHCII making it difficult to definitely identify which trimer is actually migrating during state transitions (Minagawa, 2011).

Another difference between the two light systems relates to the metabolic state of the plants in state 1. The original observations describing state 1–state 2 transitions trace back to two studies on photosynthesis in algae; both done in the light (Bonaventura and Myers, 1969; Murata, 1969). The original definition of state 1 and state 2, thus, refers to an illuminated state of the organism. A dark condition at the end of the night may exhibit the same low phosphorylation state/pattern of the LHCII as under PSI-light, but it is not identical with respect to the metabolism of the organism; e.g., the redox state of the thioredoxin pool controlling enzyme activities in the Calvin-Benson cycle (Dietz and Pfannschmidt, 2011). This is partly reminiscent to the induction of state transitions in *Chlamydomonas reinhardtii* where state 1 and state 2 represent two different metabolic states (Wollman, 2001). In *Arabidopsis* (as in all green plants) a shift from dark to light conditions represents a shift from heterotrophic to autotrophic life style (Smith and Stitt, 2007) while a PSI-light/PSII-light shift represents a shift under autotrophic conditions between two different light harvesting states. In the first case energy resources collected in the previous light phase are consumed and the light harvesting machinery is shut off while in the second case energy from photosynthesis is used and the light harvesting machinery works with two different efficiencies leading to two distinct light-adapted metabolic states (Bräutigam et al., 2009). For the degree of LHCII phosphorylation (and thus for redox-controlled STN7 kinase activity) this appears to be of no major difference; however, we observed a significant difference in PSII core phosphorylation between dark and PSI-light conditions (Figure 2). This specific difference might be a cause for the different PSII super-complex formation in these two conditions and, thus, could potentially influence the number of LHCII trimers available for the state transition process. It, therefore, is possible that metabolic activities in the dark support to some extent selective thylakoid membrane protein phosphorylation (and possibly the activity of a corresponding kinase activity). In fact, such dark phosphorylation states have been observed. CP43 was reported to be more phosphorylated in the dark than in the light (Fristedt et al., 2010) being in agreement with these observations, however, the responsible kinase activity remains to be elucidated. Our observations are also compatible with a recent study investigating the association of LHCII to PSI in dark-acclimated plants (Chukhutsina et al., 2020). This study revealed that in several plant species LHCII appears to be part of the LHCI-PSI complex in the dark. It was concluded that the plants are in a state between state 1 and state 2 because Lhcb2 is partly phosphorylated. This is consistent with our finding that LHCII at the end of the night still exhibits partial phosphorylation (Figure 2), suggesting that a complete state 1 is reached only under conditions when electrons are actively withdrawn from the PQ pool, e.g., by far-red or PSI-light illumination (Figures 1, 2). Whether a dark-induced state 1 is different from the corresponding light-induced state 1 (e.g., in terms of LHCII trimers involved) or simply remains incomplete needs to be further investigated. However, comparison of total LHCII phosphorylation (Figure 2) with LHCII trimer phosphorylation (Figure 3A) suggests a potentially higher phosphorylation state of free LHCII monomers under these conditions arguing for two different types of state 1 (Figure 5).

In sum, our study reveals that growth light set-ups with highly similar physiological effects and only minor technically different properties may still induce differences in photosynthetic acclimation responses which can be of importance for our understanding of the studied processes. The question of comparability of results between different illumination set-ups may become even more urgent with the advent of LED based illumination systems that are increasingly used now for plant growth (Seiler et al., 2017; Matsuda et al., 2021). A recent study investigated wavelength-specific effects on the redox state of the PQ pool using monochromatic LED illumination of different colors (Mattila et al., 2020). The authors observed varying efficiencies within the 400–700 nm range. These variations may explain the differences between various artificial light sources with divergent light emission spectra. The WL and PSII-light used in this study are highly similar in the range of 580–700 nm and reduce both the PQ pool with high efficiency as visible by the high LHCII phosphorylation (e.g., Figures 2, 3). The orange filter of the PSII-light, however, cuts off green and especially blue light wavelengths (Supplementary Figure S1) that may be responsible for the differences in the PS super-complex phosphorylation through the action of additional enzyme activities (see above). This will be investigated in more detail in future. In consequence, a very detailed description of the used growth light system and its spectral qualities will become mandatory for future studies on photosynthetic acclimation responses.

## Data Availability Statement

The original contributions presented in the study are included in the article/Supplementary Material, further inquiries can be directed to the corresponding author.

## Author Contributions

TP designed the research. EH, ML, and SO performed the research. All authors discussed the data and contributed to the writing of the article.

## Conflict of Interest

The authors declare that the research was conducted in the absence of any commercial or financial relationships that could be construed as a potential conflict of interest.

## Publisher’s Note

All claims expressed in this article are solely those of the authors and do not necessarily represent those of their affiliated organizations, or those of the publisher, the editors and the reviewers. Any product that may be evaluated in this article, or claim that may be made by its manufacturer, is not guaranteed or endorsed by the publisher.
