# Supplementary figures and images for: Effectiveness of Light-Quality and Dark-White Growth Light Shifts in Short-Term Light Acclimation of Photosynthesis in Arabidopsis
Source: Front Plant Sci. 2022 Jan 3;12:615253. doi: 10.3389/fpls.2021.615253 (PMC8761940; doi:10.3389/fpls.2021.615253)

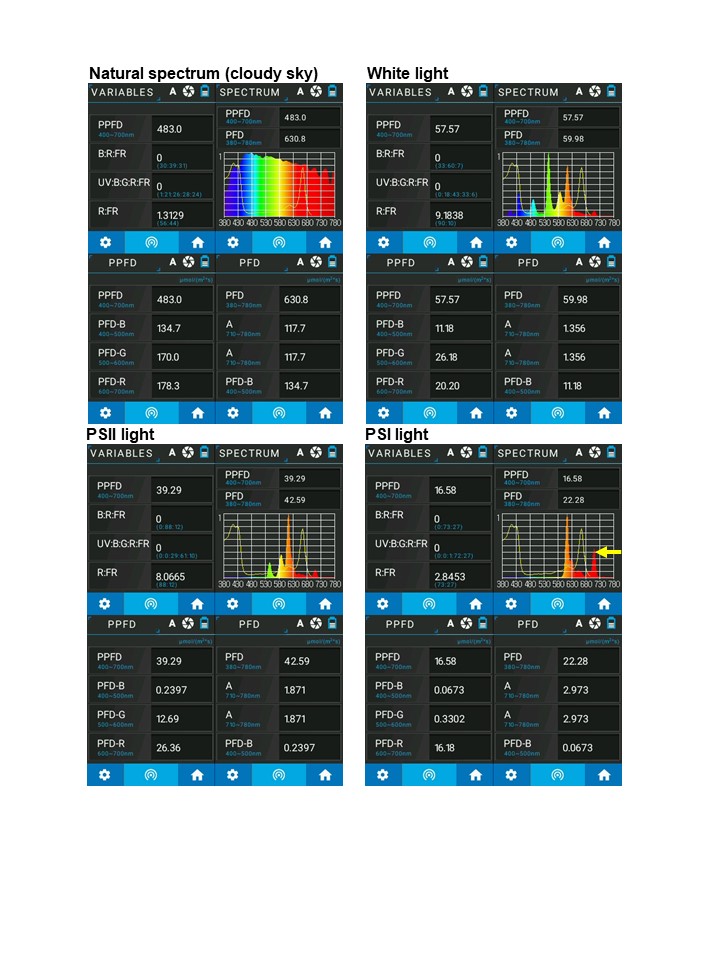

Supplement: Supplementary file 1 [file Image_1.JPEG]

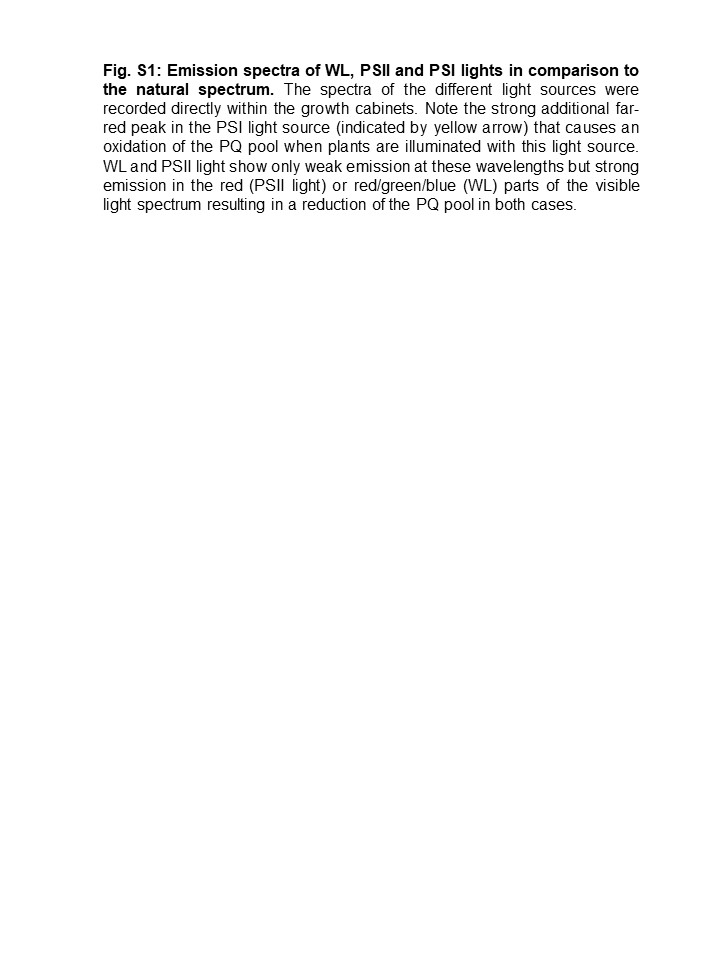

Supplement: Supplementary file 2 [file Image_2.JPEG]

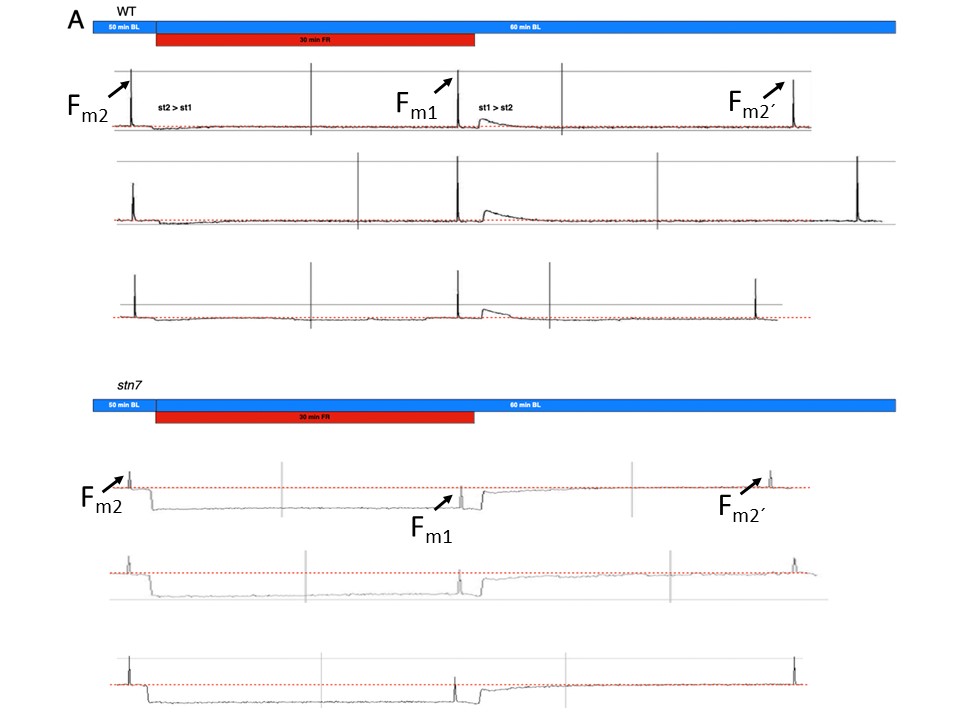

Supplement: Supplementary file 3 [file Image_3.JPEG]

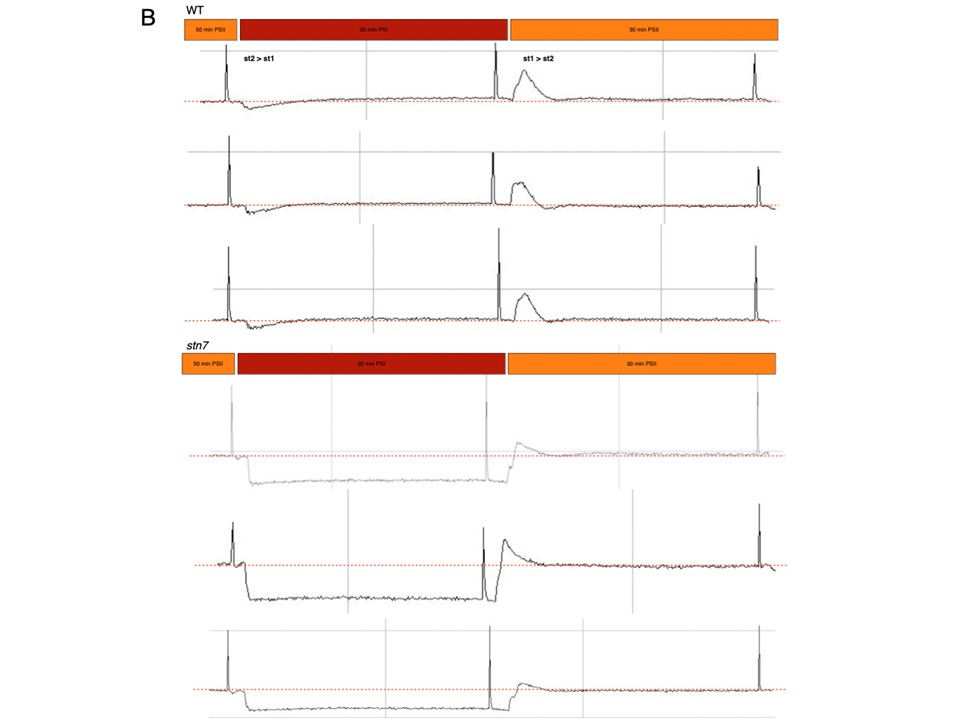

Supplement: Supplementary file 4 [file Image_4.JPEG]

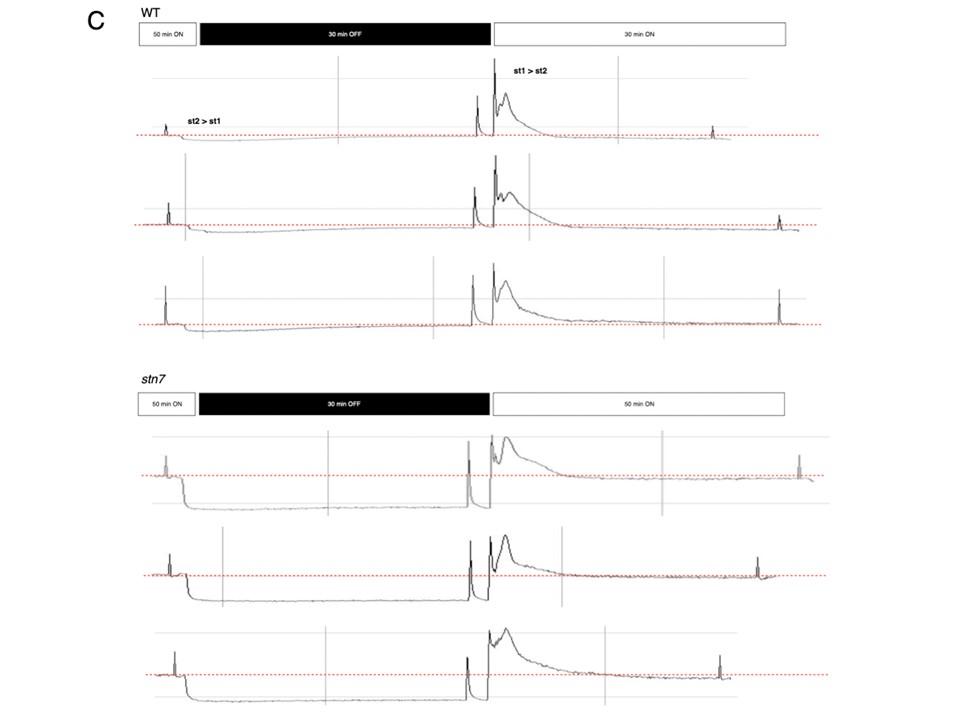

Supplement: Supplementary file 5 [file Image_5.JPEG]

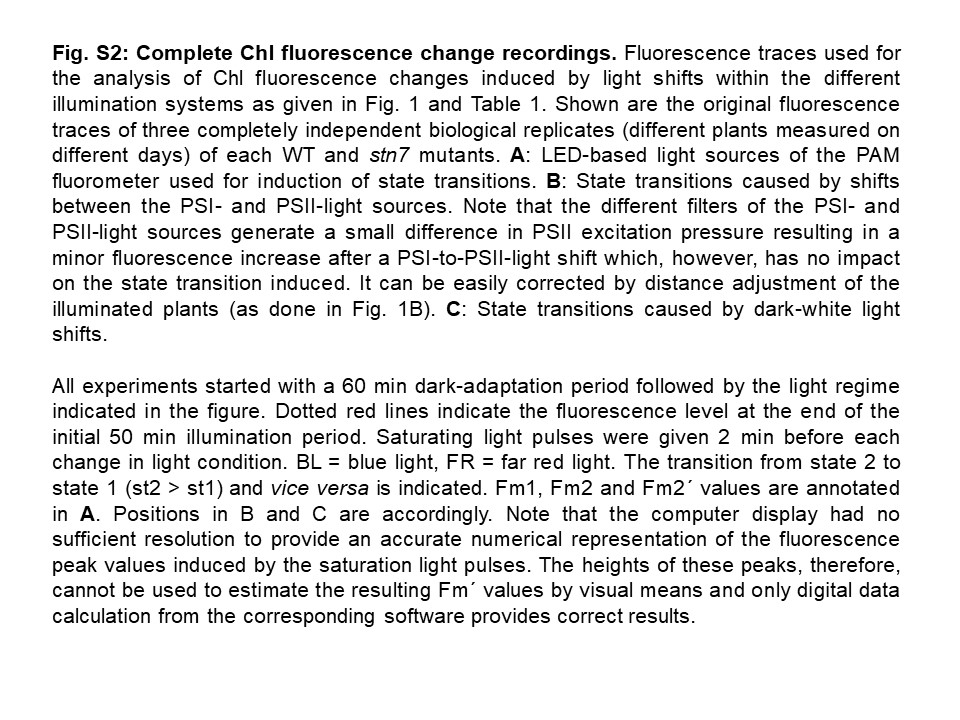

Supplement: Supplementary file 6 [file Image_6.JPEG]

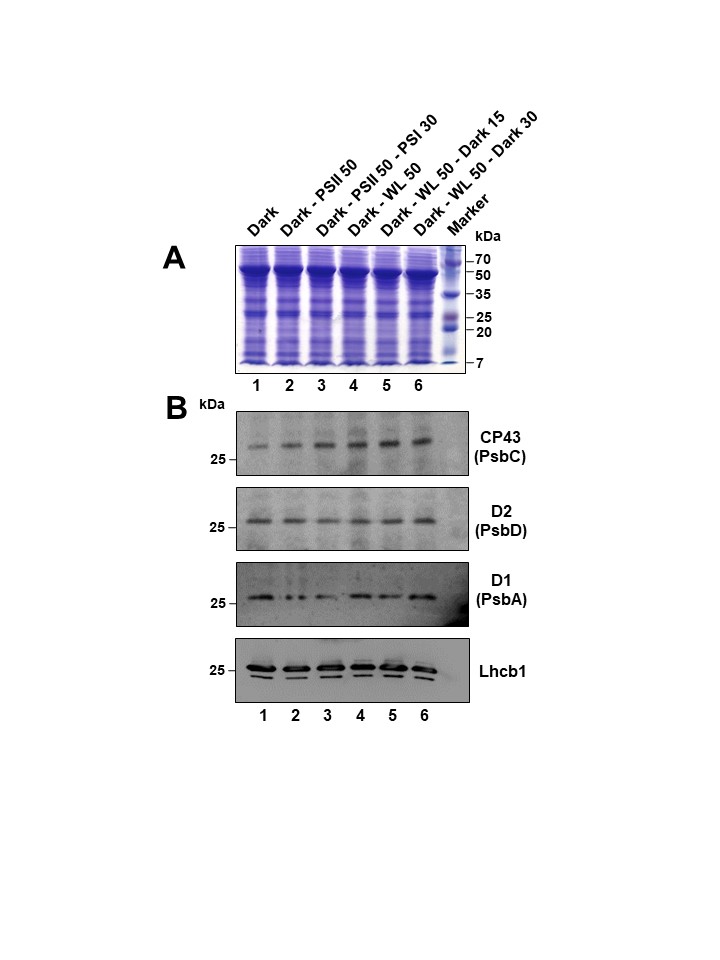

Supplement: Supplementary file 7 [file Image_7.JPEG]

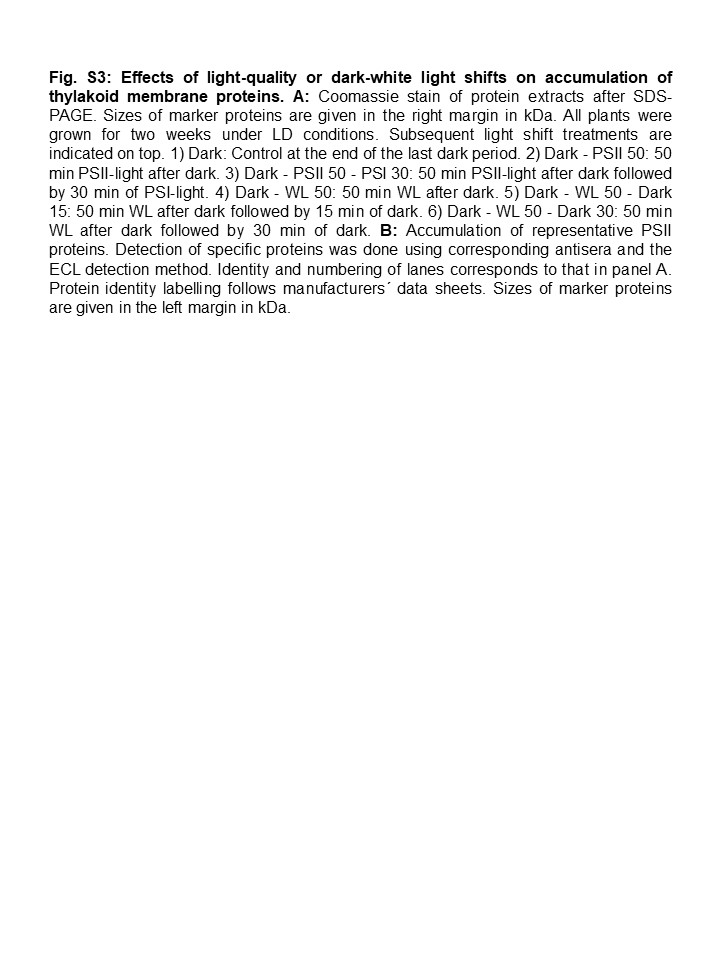

Supplement: Supplementary file 8 [file Image_8.JPEG]
