# Supplementary material for: Effectiveness of Light-Quality and Dark-White Growth Light Shifts in Short-Term Light Acclimation of Photosynthesis in Arabidopsis
Source: Front Plant Sci. 2022 Jan 3;12:615253. doi: 10.3389/fpls.2021.615253 (PMC8761940; doi:10.3389/fpls.2021.615253)
Supplement: Supplementary file 9 [file Table_1.DOCX]

**Table S1: Approximation of phosphorylation state of PSII super-complexes (PS SC) and LHCII trimers in Dark- WL 50 vs. Dark samples.** Signal intensities of indicated bands were determined with ImageJ software (after subtraction of background signals) and expressed as relative values. Indications SC1-3 are given in both figures. AVG: Average of the three PSII super-complexes. SD: Standard deviation. N.D.: non detectable.

| **Band** | **Intensity**  **Dark – WL 50 *vs.* Dark (%)** | **AVG** | **SD** | **Intensity**  **Dark – WL 50 *vs.* Dark (%)** | **AVG** | **SD** | **Phosphorylation state (%)**  **Dark – WL 50 *vs.* Dark** |
| --- | --- | --- | --- | --- | --- | --- | --- |
|  | Phosphorylation signal Fig. 3 | | | Coomassie signal Fig. 4 | | |  |
| **PSII SC1** | 49.5 | 63 | 25 | 445 | 322 | 110 | 11 |
| **PSII SC2** | 48.5 |  |  | 291 |  |  | 16 |
| **PSII SC3** | 91.6 |  |  | 231 |  |  | 39 |
| **LHCII trimers** | 219 | n.d. | n.d. | 91 | n.d. | n.d. | 241 |
